# A Two-Stage Deep Generative Model for Masked Face Synthesis

**DOI:** 10.3390/s22207903

**Published:** 2022-10-17

**Authors:** Seungho Lee

**Affiliations:** Department of Future Technology, Korea University of Technology and Education, Cheonan-si 31253, Chungcheongnam-do, Korea; leesh903@koreatech.ac.kr

**Keywords:** convolutional auto-encoder (CAE), facial pose estimation, masked face generation, masked face synthesis, deep generative model

## Abstract

Research on face recognition with masked faces has been increasingly important due to the prolonged COVID-19 pandemic. To make face recognition practical and robust, a large amount of face image data should be acquired for training purposes. However, it is difficult to obtain masked face images for each human subject. To cope with this difficulty, this paper proposes a simple yet practical method to synthesize a realistic masked face for an unseen face image. For this, a cascade of two convolutional auto-encoders (CAEs) has been designed. The former CAE generates a pose-alike face wearing a mask pattern, which is expected to fit the input face in terms of pose view. The output of the former CAE is readily fed into the secondary CAE for extracting a segmentation map that localizes the mask region on the face. Using the segmentation map, the mask pattern can be successfully fused with the input face by means of simple image processing techniques. The proposed method relies on face appearance reconstruction without any facial landmark detection or localization techniques. Extensive experiments with the GTAV Face database and Labeled Faces in the Wild (LFW) database show that the two complementary generators could rapidly and accurately produce synthetic faces even for challenging input faces (e.g., low-resolution face of 25 × 25 pixels with out-of-plane rotations).

## 1. Introduction

Face recognition (FR) is one of the most important problems in computer vision area as it has a wide range of real-world applications. Conventional FR functions are generally performed with primary facial features such as eyes, nose, and mouth, assuming non-occluded faces [1]. However, one may encounter situations where faces are largely occluded with unwanted objects such as accessories. Facial occlusion is one of the most challenging problems in FR because the occluded parts on the human face can be arbitrary in position, size and shape [2,3]. Due to the prolonged COVID-19 pandemic, wearing masks has been recommended in many countries to avoid spreading of the virus. Masked face recognition (MFR) is a special case of occluded FR [3]. It has been known that MFR is very challenging because the features of mouth and nose are severely damaged; thus, the discriminating features are greatly reduced [3]. To address the challenges of MFR, some research efforts with deep learning approaches have been made [4,5]. One of the most straightforward methods for MFR is discarding occluded face region. In general, this approach first detects the occluded regions and discard them as preprocessing [4,6]. The authors in [4] have proposed to apply three pre-trained deep convolutional neural networks (CNNs) [7,8], called VGG-16 [9], AlexNet [7], and ResNet-50 [10], aiming to extract deep features from the remaining facial regions (mostly eyes and forehead regions). The main drawback of the occlusion discarding based approach is limited discriminating capability. The authors in [5] have proposed a deep learning method based on Generative Adversarial Network (GAN) [11] that removes the mask region and reconstructs the missing region with synthetic facial appearance. They have tried to preserve structural and shape consistency in the recovered face using two discriminators where one seeks to learn the global structure of the face and then the other focuses on learning deep missing regions (such as mouth and teeth regions which are far away from the occlusion boundary caused by wearing the mask) [5].

MFR methods often need large datasets with masked face images to make full use of the learning capability of deep learning technologies. To address this issue, the authors in [12] have constructed Real-world Masked Face Recognition Dataset (RMFRD) by collecting 2203 images of 525 subjects wearing masks, and 90,000 images of the same 525 subjects without masks [12]. However, in this dataset, face image pairs of with and without mask are not available because the collected face images are basically real-world images. In order to expand the volume and diversity of the masked face dataset, the authors in [12] have generated synthetic images of masked faces in the existing public large-scale datasets (i.e., LFW [13] and Webface [14] datasets). As a result, this simulated datasets (called SMFRD) contain about 500,000 face images of 10,000 subjects. To improve the data manipulation efficiency during the construction of SMFRD dataset, an automatic mask wearing software based on Dlib library [15] has been utilized. Similarly, in [16], 68 facial landmarks are detected using Dlib library and various synthetic masks are generated by connecting the points which form a polygon filled with a color. In [17], a Dlib based face landmark detector is used to identify the face tilt and six key features for applying mask. According to the face tilt, a template mask is selected from the mask library. Next, the selected template mask is warped based on the key features to fit properly the face appearance. Such synthetically masked face images [12,16,17] are advantageous over real masked images in a sense that the synthetic face images can be used along with their original (i.e., unmasked) face images. For example, face image pairs (masked/unmasked) are required in the training stage of deep networks for unmasking of masked face and filling the missing region. In addition, the face image pairs can be useful for investigating the effect of FR performance degradation caused by wearing masks. However, the geometric based methods in [12,16,17] that rely on landmark localization could suffer from low resolution or occlusion in real-world face images.

To cope with the aforementioned limitations, this paper proposes a compact and practical method to synthesize masked face. The main contributions of this paper are twofold:

1. A two-stage generative model based on cascading two convolutional auto-encoders (CAEs) [18] is introduced in this paper. The goal of the former CAE is to obtain a virtual mask pattern suitable for an input face image in terms of pose view. The latter CAE aims to generate a segmentation map to localize the mask pattern region. Based on the segmentation map, the mask pattern can be successfully fused with the input face by means of simple image processing techniques (e.g., pixel-wise sum). Different from the methods in [12,16,17], the proposed generative model relies on face appearance reconstruction without any facial landmark detection or localization techniques.
2. With the appearance-based approach described above, the proposed generative model can be practically used for constructing large datasets of masked face images. As demonstrated in experiments, the proposed method is able to process low resolution faces of 25 × 25 pixels with acceptable synthesis results without high loss in recognition rate (refer to Section 4.3). Additionally, the proposed method works well with moderately rotated face images. It is possible to complete learning of the two complementary CAEs with tens of seconds using a PC with a single GPU (refer to Table 1). Hence, one could easily extend the cascaded network for applying various mask patterns.

The rest of this paper is organized as follows. Section 2 presents an overview of the proposed method. Section 3 describes in detail the cascading of CAEs to obtain a synthetic masked face. Section 4 presents experimental results to demonstrate the robustness and efficiency of the proposed method. The conclusion is drawn in Section 5.

## 2. Overview of Proposed Method

**Figure 1:**
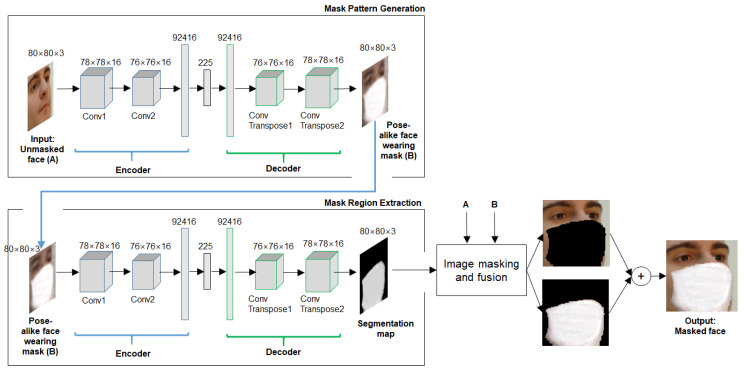
An overall framework of the proposed method. In this figure, an RGB face image of 80 × 80 pixels is used for input as an example. The stage of mask pattern generation aims to produce pose-alike face with a mask pattern reflecting the pose view of an input unmasked face. The stage of mask region extraction focuses on localizing the mask pattern by producing the segmentation map. In the stage of image masking and fusion, the mask pattern was fused with the input unmasked face, resulting in a realistic masked face.

**Figure 2:**
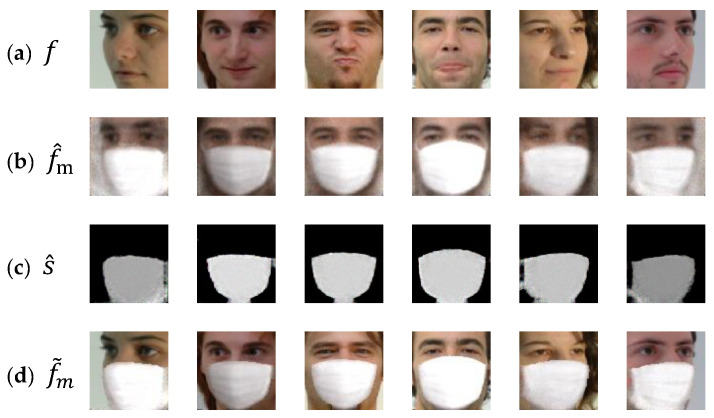
(**a**) Input of the generator $G_{p}$. (**b**) Output of the generator $G_{p}$. (**c**) Output of the generator $G_{r}$. (**d**) The synthesized face.

The proposed method aims to transfer an unmasked face image (input) to a masked face image (output). Specifically, for a given unmasked face image, a masked face image is generated by overlaying a fake mask pattern on the corresponding face. The proposed method contains two sequential stages, each of which utilizes a CAE. Auto-encoder is a method specialized in unsupervised learning, consisting of an encoder and a decoder. The encoder learns to translate the input into an internal representation. The decoder learns to convert the internal representation into the same form as the input. The best-known neural network for image modeling is CNN which enables to effectively retain the structural information connected between pixels of an image. For this reason, CNN has been successfully applied to an auto-encoder where CNN is deployed in both the encoder and decoder. Figure 1 shows an overview of the proposed method. In the stage of mask pattern generation, the CAE takes an unmasked face image as input (e.g., Figure 2a), and produces a virtual image of face wearing a mask. For this purpose, the CAE needs to be pretrained for the source domain and target domain, which are unmasked face and masked face, respectively. Note that the difference between the two domains is whether mask is present or not. It should be noted that the main objective of this stage is to obtain a mask pattern image that accurately fits the input face in terms of position, size, and shape. To this end, the CAE is required to faithfully reconstruct a pose-alike face for the input face image. In this paper, the face detection function in the Dlib library is used to detect face region. The input face image is obtained by aligning the detected face region based on the two eye-coordinates. This allows the CAE to mainly focus on the out-of-plane rotation (pitch or yaw) during reconstruction of a pose-alike face while the effect of the in-plane rotation (roll) could be suppressed.

In the stage of mask region extraction, the secondary CAE takes the output of the masked pattern generation and predicts a segmentation map which localizes the mask region on the face. As illustrated in Figure 1, the bright region corresponds to the mask region. The segmentation map aims to fuse the mask pattern with the input face for the purpose of mask overlay on face. Similar to the previous stage, the CAE needs a pretraining step for two different domains which are masked face and its segmentation map indicating the mask region, respectively. The inputs (i.e., the unmasked face and the pose-alike face wearing a mask) to the two CAEs and the output (i.e., segmentation map) of the secondary CAE are readily fed into the image masking and fusion stage which combines the unmasked face and the mask pattern, resulting in a realistic face wearing mask (e.g., Figure 2d). Details on the proposed method for the masked face synthesis will be given in Section 3.

## 3. Masked Face Synthesis Using the Proposed Generative Model

### 3.1. Mask Pattern Generation

This section describes in detail the mask pattern generation which is the former stage of the proposed generative model. The CAE used in this stage is called mask pattern generator (denoted by $G_{p}$). Given an input (unmasked) face f, $G_{p}$ generates a pose-alike face ${\hat{f}}_{m}$ with a mask pattern. It is expressed as

(1) $${\hat{f}}_{m}=G_{p}\left(f\right).$$

Figure 2b shows some examples of ${\hat{f}}_{m}$. As is seen, the pose view of each ${\hat{f}}_{m}$ is similar to that of the associated face f in Figure 2a. Note that, during the face generation, $G_{p}$ is likely to pay more attention to the global appearance (e.g., pose view) than the local details due to the compressed representation at the middle of the CAE.

Figure 1 shows the network architecture of $G_{p}$ used in this paper. An RGB face image of 80 × 80 pixels is converted to a cubic (Conv1) consisting of 16 feature maps of 78 × 78 pixels, which becomes another smaller cubic (Conv2). These convolution layers are designed to retain the spatial relationships in the image data. After the encoding process, in the middle, there is a fully connected auto-encoder and its hidden layer consists of 225 nodes. The 225 nodes are called pose vector in this paper because those are expected to encode pose-related information. The decoder is symmetric to the encoder in terms of layer structure.

**Figure 3:**
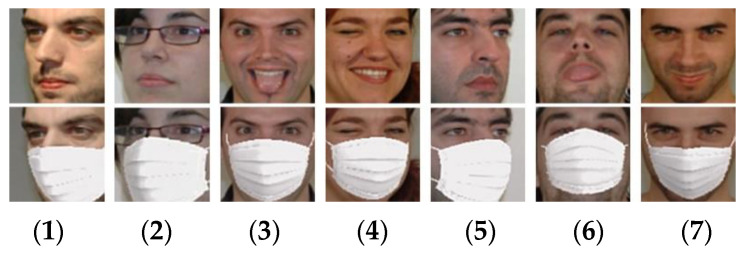
Image pairs of unmasked face and masked face used for learning the mask pattern generator. The masked face images are obtained by using the SNOW application. The pose views of the used faces can be roughly categorized into seven groups (left-to-right): (**1**) −45~60 degrees; (**2**) −15~30 degrees; (**3**) frontal; (**4**) +15~30 degrees; (**5**) +45~60 degrees; (**6**) up; and (**7**) down.

In order to learn $G_{p}$, let $f_{}^{\left(i\right)}$ denotes the *i*-th unmasked face (source domain) and $f_{m}^{\left(i\right)}$ denotes the corresponding masked face (target domain). Note that i is index of a face image pair ($f_{}^{\left(i\right)}$ and $f_{m}^{\left(i\right)}$) contained in the training set. To guarantee that the only difference between $f_{}^{\left(i\right)}$ and $f_{m}^{\left(i\right)}$ is the presence of mask, it is possible to create a synthetic face $f_{m}^{\left(i\right)}$ by overaying a virtual mask pattern on $f_{}^{\left(i\right)}$ with a manual process (e.g., using Adobe Photoshop) or an automatic software tool. In this paper, a smart phone camera application called SNOW [19] is used for automatically producing the synthetic masked faces. The application is characterized by virtual stickers using augmented reality and photographic filters [19]. Figure 3 shows some image pairs (masked/unmasked) used for learning $G_{p}.$ To construct the training set, face image pairs have been collected from GTAV Face database [20]. Various facial pose views are included in the training set to deal with different pose views when generating mask patterns. The used faces can be roughly categorized into seven groups as shown in Figure 3. To learn $G_{p}$, the loss function *L*_p_ is defined as follows:

(2) $$L_{p}=\frac{1}{N}\sum_{i=1}^{N}MSE(f_{m}^{\left(i\right)},\;{\hat{f}}_{m}^{\left(i\right)}),$$

where $MSE\left(f_{m}^{\left(i\right)}\;,\;{\hat{f}}_{m}^{\left(i\right)}\right)$ is the mean squared error [21] which computes the average of the squares for the pixel differences between the *i*-th target masked face $f_{m}^{\left(i\right)}$ and the *i*-th pose-alike faces denoted by ${\hat{f}}_{m}^{\left(i\right)}\;$(i.e., output of $G_{p}$ for $f_{}^{\left(i\right)}$). N is the size of a mini batch.

### 3.2. Mask Region Extraction

As shown in Figure 2a,b the identity-related appearance in each masked face ${\hat{f}}_{m}$ is clearly different from that in the input face f. This is due to the fact that if a subject in f is not present during the learning of $G_{p},$ a conventional CAE is not capable of faithfully reconstructing the identity-related appearances. Furthermore, ${\hat{f}}_{m}$ looks very blurry. To cope with the aforementioned problems, the generated mask pattern is fused with the input face f in the proposed method. In order to obtain the only mask pattern region from ${\hat{f}}_{m},$ this section describes the mask region extraction which is the latter stage of the generative model. The CAE used in this stage is called mask region extractor and denoted by $G_{r}$. During the inference (or prediction), the input to $G_{r}$ is ${\hat{f}}_{m}$ which is the output of the $G_{p}.$ Then, $G_{r}$ produces a segmentation map $\hat{s}$ as

(3) $$\hat{s}=G_{r}\left({\hat{f}}_{m}\right),$$

where $\hat{s}$ aims to localize the mask region in ${\hat{f}}_{m}$. Figure 2c shows some example images for $\hat{s}$. As illustrated in Figure 1, the mask region extractor is the same as the mask pattern generator in terms of network architecture.

**Figure 4:**
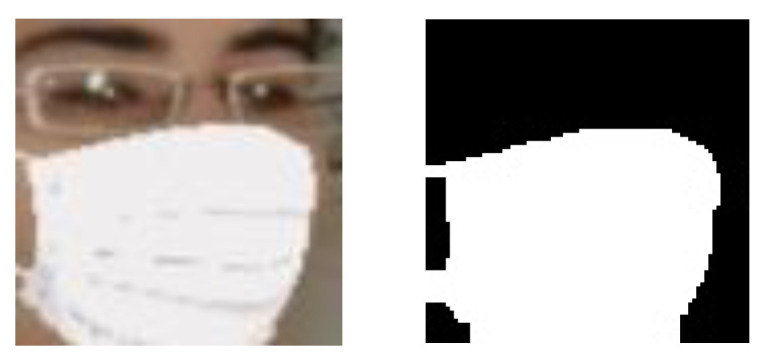
Examples for a pair of masked face image (**left**) and segmentation map image (**right**) that can be used to learn $G_{r}$.

To learn $G_{r}$, let $s_{}^{\left(j\right)}$ denotes the segmentation map (target domain) associated with the *j*-th masked face $f_{m}^{\left(j\right)}$ (source domain). j is index of a pair ($f_{m}^{\left(j\right)}$ and $s_{}^{\left(j\right)}$) used as training data. The segmentation map images have been manually created by the author, each of which is a binary image. Figure 4 shows examples of a masked face $f_{m}^{\left(j\right)}$ and its segmentation map $s_{}^{\left(j\right)}$, which can be used as training data. Similar to $G_{p},$ the mean squared error is used to define the loss function *L*_r_ for learning $G_{r}$ as follows:

(4) $$L_{r}=\frac{1}{M}\sum_{j=1}^{M}MSE(s_{}^{\left(j\right)},\;{\hat{s}}_{}^{\left(j\right)}),$$

where ${\hat{s}}_{}^{\left(j\right)}$ denotes the output of $G_{r}$ for $f_{m}^{\left(j\right)}.$ M is the size of a mini batch.

### 3.3. Image Masking and Fusion

**Figure 5:**
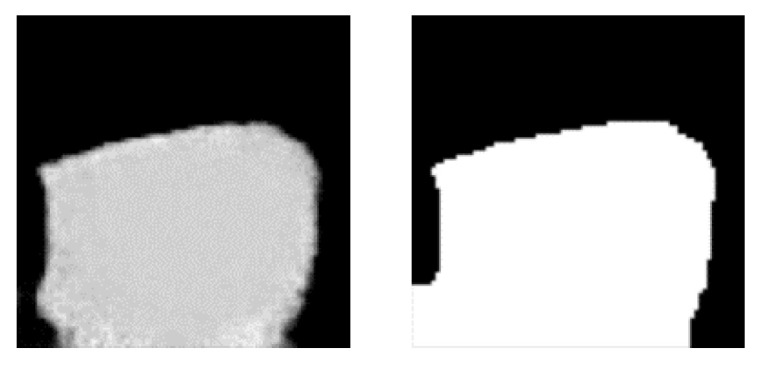
Examples for a segmentation map $\hat{s}\;$(**left**) and the processed binary segmentation map ${\hat{s}}_{b}$ (**right**).

Because each segmentation map $\hat{s}$ generated using (3) is not a binary image as shown in Figure 5, it cannot be directly used for the purpose of fusing mask pattern with input face. Thus, it is converted into a binary image by using grayscale transformation and binarization. To make the binary segmentation map clean, it is processed via simple image morphology operations. Specifically, erosion operation is first applied, which is followed by dilation operation. Figure 5 shows an example of segmentation map denoted by ${\hat{s}}_{b}$. Then, the synthetic masked face ${\tilde{f}}_{m}$ can be obtained by using the following operations:

(5) $${\tilde{f}}_{m}\;=\;BA({\hat{s}}_{\mathrm{binv}},\cdot f)\;⨁\;BA({\hat{s}}_{b},\cdot {\hat{f}}_{m}),$$

where *BA* is bitwise and operation used for image masking and ⨁ is image sum operation. ${\hat{s}}_{\mathrm{binv}}$ is the grayscale inverted image of ${\hat{s}}_{b}.$

## 4. Experiment

This section presents extensive experiments for the proposed method using Labeled Faces in the Wild (LFW) database [13] and GTAV Face database. To learn the mask pattern generator $G_{p}$ and the mask region extractor $G_{r}$, Adam [22] was used as optimizer and epoch was set to 100.

### 4.1. Results for Various Pose Views and Resolutions

**Figure 6:**
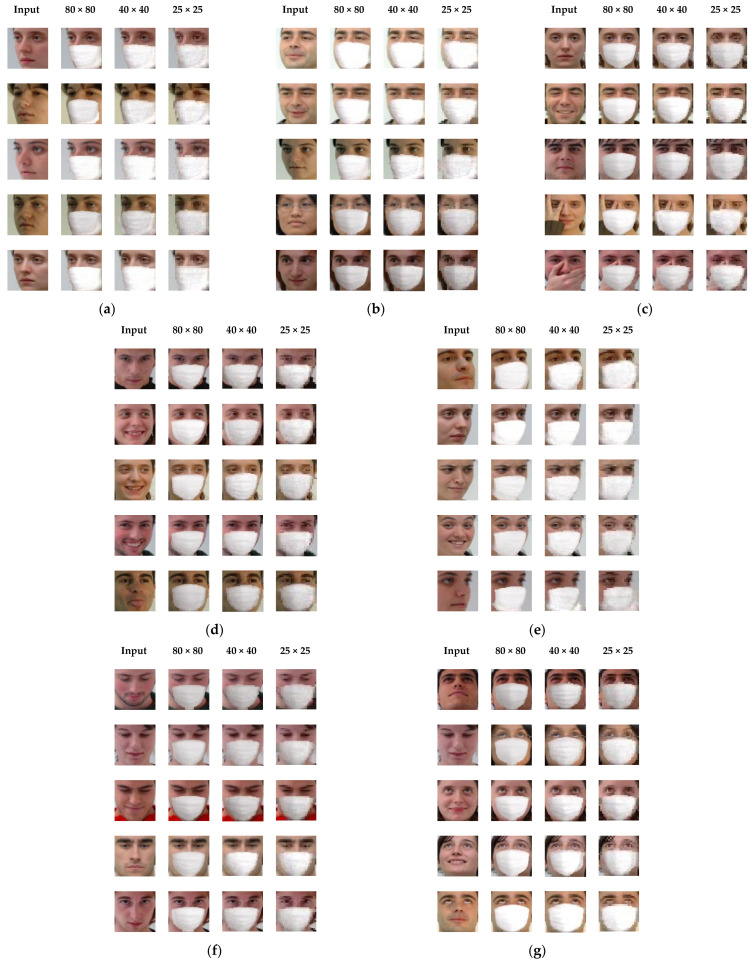
Results for different facial poses and resolutions. (**a**) −45~60 degrees. (**b**) −15~30 degrees. (**c**) frontal. (**d**) +15~30 degrees. (**e**) +45~60 degrees. (**f**) down. (**g**) up.

This section aimed to investigate the effectiveness of the proposed method under different pose views and resolutions of input faces. GTAV Face database was used in this experiment. The training set consisted of 903 images (i.e., 301 unmasked faces, 301 masked faces, and 301 segmentation maps) from 22 (out of 44) subjects, and the testing set consisted of 397 unmasked face images from the remaining 22 subjects. Seven different pose views were considered: (1) −45~60 degrees; (2) −15~30 degrees; (3) frontal; (4) +15~30 degrees; (5) +45~60 degrees (6) down; and (7) up. For the case of facial resolution, three different pixel sizes were used: (1) 80 × 80; (2) 40 × 40; and (3) 25 × 25. Furthermore, 80 × 80 pixel images were downsampled to obtain 40 × 40 and pixel images. From the results of the masked face synthesis illustrated in Figure 6, the following observations were made:

(1) The proposed method was able to generate similar mask synthesis results for the three different facial resolutions of an input face image. This demonstrated that the proposed method was robust to variation in facial resolution.
(2) The generated mask pattern fitted accurately the faces with moderate out-of-plane rotations in pitch or yaw. This was because the generator $G_{p}$ could be learned to reconstruct pose-alike faces (refer to Section 4.2) by utilizing training faces with different pose views.
(3) Thanks to the reconstruction ability of the appearance-based method, the proposed model was able to generate the masked faces even for the faces occluded by a hand (see Figure 6c).

Table 1 shows the computation time required for the learning and inference. Because the two generators could be learned within one minute, one could immediately retrain them for masked face synthesis by applying different mask patterns. Using the learned networks, it took only 0.28 s to process 397 face images. This result demonstrated the low computation cost of the proposed metho.

### 4.2. Analysis on Facial Pose

**Figure 7:**
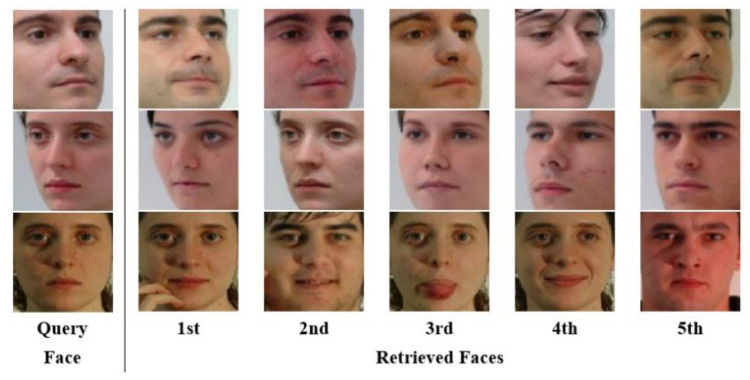
Face retrieval result. Faces were sorted in ascending order by pose vector distance (i.e., the smallest Euclidean distance value is ranked as 1st).

The goal of this experiment was to investigate that the encoded pose vector in $G_{p}$ was useful for reconstructing similar faces to an input face in terms of pose view. As shown in Figure 7, three face images with different pose views were selected as the query faces from the testing set described in Section 4.1. Next, the encoded pose vector of each query face image was compared with those of the remaining face images within the testing set. Using the Euclidean distance, the most similar faces to the query faces were retrieved. From the result in Figure 7, one could see that the retrieved faces looked similar in pose view to the query face. This demonstrated that a pose vector contained pose-related information that could be useful for successfully generating a pose-alike face.

### 4.3. Analysis on Facial Resolution

**Figure 8:**
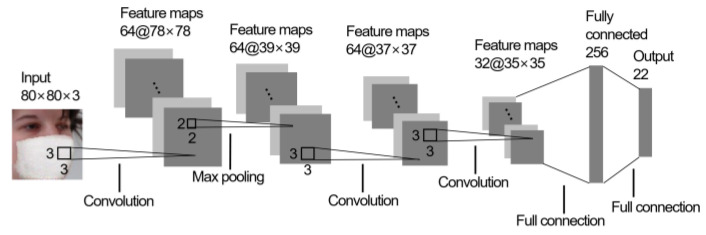
The CNN classifier used for measuring the FR rates. A face image of 80 × 80 pixels was used as an input image in this figure.

This section aimed to quantitatively verify that the proposed generative model was robust against variation in facial resolution. For this, the FR accuracies (in recognition rate) were measured for the three different facial resolutions present in Figure 6. Similar to the method described in Section 4.1, 80 × 80 pixel images were downsampled to obtain 40 × 40 and 25 × 25 pixel images. After that, the resized face images were fed to the two-stage generator consisting of $G_{p}$ and $G_{r}$ in order to synthesize the masked face images. For each facial resolution, a total of 397 masked face images were obtained by applying the proposed method to 397 unmasked face images in the testing set described in Section 4.1. Five-fold cross validation was adopted to measure the recognition rates. Note that the recognition rates measured for the unmasked face images (e.g., face images of the ‘Input’ columns in Figure 6) were also included for comparison. As illustrated in Figure 8, a conventional CNN classifier was employed for measuring the facial recognition rates. The first convolution layer used 64 filters with sizes of 3 × 3 which was followed by a 2 × 2 max pooling layer to reduce the spatial size of the feature maps and computational cost of the network. Next, the second and third convolution layers employed 64 filters and 32 filters, respectively with sizes of 3 × 3. A fully connected layer was included at the end of the CNN network to classify subject class. The output layer had a total of 22 nodes corresponding to 22 subject classes. In order to learn the CNN model, epoch and batch size were set to 100 and 5, respectively. Adam was used as optimizer.

**Figure 9:**
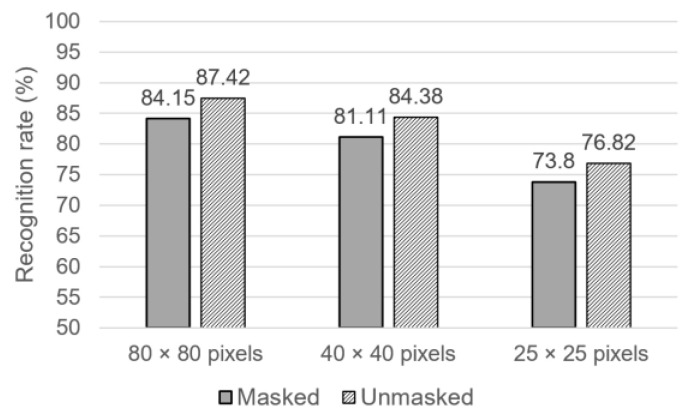
Recognition rates for different facial resolutions. The recognition rates measured for unmasked face images were also present for comparison.

The recognition rates for the three facial resolutions were illustrated in Figure 9. The average performance gaps between the masked face images and the unmasked face images were around 3%. It was worth noting that the performance gap was maintained for face images with low resolution (i.e., 25 × 25 pixels).

To further investigate the effect of using different facial resolutions of masked face synthesis on recognition rate, the masked face images generated from the unmasked face images of 80 × 80 pixels were resized to 25 × 25 pixels and their recognition rate was measured. The recognition rate was 73.54% which was very similar with the result (73.80%) for the masked face images generated from the unmasked face images of 25 × 25 pixels (see Table 2). These results demonstrated that the proposed generative model was able to preserve discriminative information (i.e., identity-related facial appearance) even when synthesizing masked face at low-resolution.

### 4.4. Results on Real-World Face Images (LFW Database)

In this section, the effectiveness of the proposed method was investigated using another dataset called LFW. LFW was a database of face photographs designed for studying the problem of unconstrained face recognition, which contained real-world face images of faces collected from the web. LFW database was much more challenging than GTAV Face database due to the uncontrolled conditions of the face acquisition. To consolidate training data, in addition to the original training set consisting of 903 images, 397 faces (i.e., testing set in Section 4.1) were also included in the construction of a new training set. To this end, a total of 1191 images (i.e., 397 unmasked faces, 397 masked faces, and 397 segmentation maps) were obtained. To make the new training set more complete in terms of face pose view, 2094 (=1191 + 903) images were horizontally flipped. As a result, a total of 4188 images were used as the training data.

**Figure 10:**
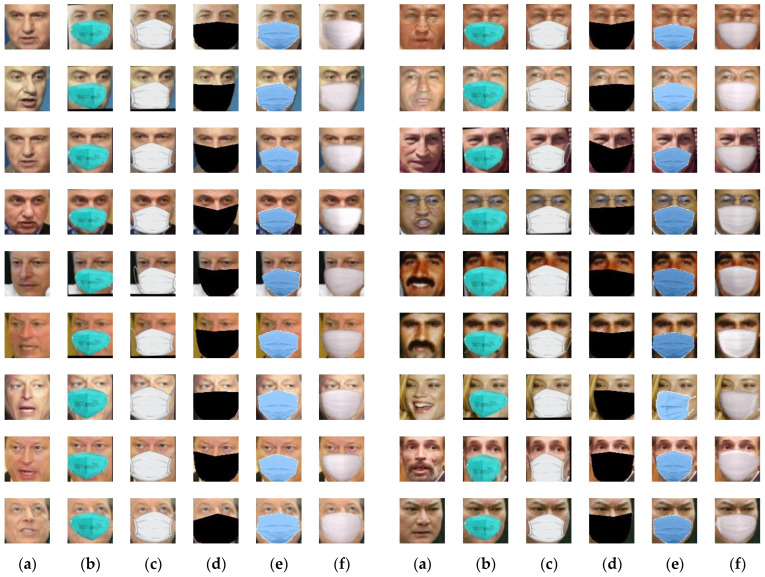
Comparison results on LFW database. (**a**) Input. (**b**) Method in [12]. (**c**) Method in [12]. (**d**) Method in [16]. (**e**) Method in [17]. (**f**) Proposed method.

Three geometric based methods [12,16,17] were present in this section for comparison purposes where the source codes provided by the authors were used to obtain reliable results. For the comparison methods and the proposed method, face images of 80 × 80 pixels were used as input. Figure 10 illustrates the comparison results on a number of face images from LFW database. For the case of [12], the synthetic faces looked unrealistic because they had cartoon-like mask patterns. It was shown in Figure 10d that the mask patterns obtained by using the method in [16] looked binary masking maps due to the very simple algorithm (i.e., connecting the points which formed a polygon filled with a color). The method in [17] achieved better-fitting mask patterns by warping the mask according to estimated mask key points. However, due to the warping of a two-dimensional mask pattern image, one might feel the small gaps between the face and the mask pattern (see the fifth and sixth face images in the fifth column of Figure 10). The proposed method was comparable with the method in [17]. Because the proposed method was based on generating a pose-alike face wearing a mask pattern, the mask pattern could fit accurately and tightly to the input face with different pose views (see the fifth and sixth face images in the sixth column of Figure 10).

**Figure 11:**
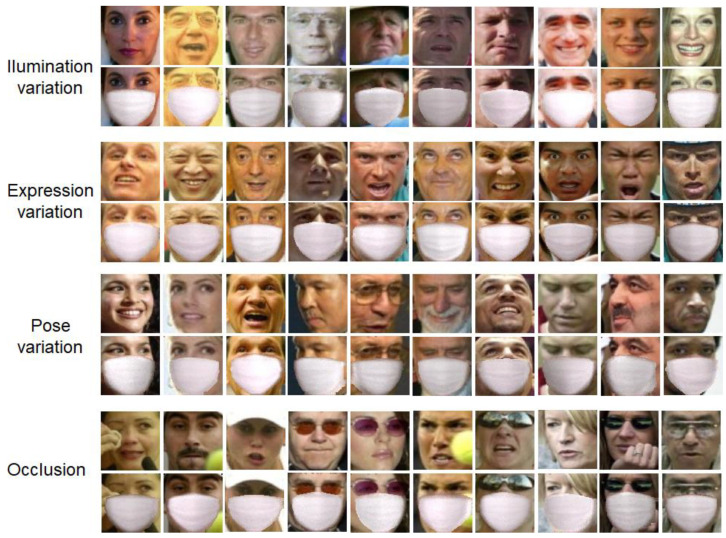
Results for the proposed masked face synthesis on LFW database. The results were categorized into four different challenges.

Figure 11 presents more results for the proposed masked face synthesis, which were categorized into four different challenges: (1) Illumination variation; (2) Expression variation; (3) Pose variation; and (4) Occlusion. For constructing the testing set, 2115 faces of 400 + celebrities were collected from the image dataset downloaded in Kaggle [23]. As shown in Figure 11, the proposed method achieved acceptable results on real-world face images. For example, the proposed method worked accurately with severe illumination conditions including various outdoor lighting environments. Furthermore, the proposed method could yield stable synthesis results even with combined challenges (see the first face in the Expression variation challenge in Figure 11, which was also characterized by out-of-plane rotation). The results for 2115 synthetic face images can be found in the author’s github [24]. It should be noted that, for the proposed method, the two generators $(G_{p}$ and $G_{r})$ were learned using the training set derived from GTAV Face database and tested on LFW database. The results of the cross-database validation demonstrated the generalization capability of the proposed generative model.

As shown in Figure 3, SNOW application was able to generate realistic masked face images. However, SNOW was not suitable for processing a large number of unmasked face images from many different subjects because it was basically designed for processing a single selected photo stored in smart phone or a video frame captured by a smart phone camera. On the other hand, the proposed generative model was able to deal with a large number of face images due to its batch processing design. Furthermore, different from SNOW which provided users with limited number of mask patterns, the proposed generative model could be extended to other various mask patterns. For example, the synthetic face images in Figure 10e could be used as training data for learning the mask pattern generator $G_{p}$.

## 5. Conclusions

This paper proposed a two-stage generative model to synthesize masked face images. The former generator aimed to produce a pose-alike face with a mask pattern reflecting the pose view of an input face image. The latter one focused on localizing the mask pattern by producing the segmentation map. Then, the mask pattern was fused with the input face, resulting in a realistic masked face.

Research on face recognition (FR) with masked faces has been becoming increasingly important due to the prolonged COVID-19 pandemic [25]. The proposed method aimed to generate a fake mask and overlay it on a real face. It was believed that the proposed method could be useful for constructing a large face recognition dataset that contained image pairs of unmasked face and synthesized masked face. Such synthetically masked face images were advantageous over real masked face images in a sense that the synthetic face images could be used along with their original (i.e., unmasked) face images. The face image pairs (masked/unmasked) could be used as training data for reducing the effect of wearing mask in MFR and used for investigating the effect of FR performance degradation caused by wearing masks.

The proposed method could be practically used for the following reasons:

(1) The generative model was very compact and easy to implement. The two generator networks did not require complicated network settings (recall that the two generators shared the same network architecture which have 16 feature maps for convolution layer and 225 nodes for hidden layer).
(2) As demonstrated in qualitative and quantitative experiments, the generative model could be robust against moderate out-of-plane rotation (up to ± 60 degrees) and resolution variation present in face images (80 × 80, 40 × 40, and 25 × 25 pixels).
(3) The generative model had very low computational cost for both training and inference. Because it took only 0.28 s to process 397 face images, the model could rapidly generate synthesized masked faces with real-time processing. Furthermore, due to the compact network architecture, the generative model could be easily extended to other mask patterns by simply retraining the generator networks.

Due to the efficiency of the proposed method, it could also be useful for research of video-based face recognition [26,27,28]. For future work, generative models to rapidly synthesize the masked faces for video sequence will be studied. In particular, a hardware format (such as Raspberry Pi with camera module) could be incorporated into the proposed method which allows collecting a large amount of real-world training image sequences of masked faces.

## Figures and Tables

**Table 1:** Computation Time For $G_{p}$ and $G_{r}$. Here, 80 × 80 was selected as facial resolution.

80 × 80 Was Selected as Facial Resolution		
Generator	Computation Time (s)	
	Learning	Inference (for Processing 397 Face Images)
*G*_p_	25.66	0.14
*G*_r_	25.04	0.14

**Table 2:** Effect of using different facial resolutions of masked face synthesis on recognition rate.

Performing Masked Face Synthesis	Performing Face Recognition	Recognition Rate (%)
at 80 × 80 pixels	at 80 × 80 pixels	84.15
at 40 × 40 pixels	at 40 × 40 pixels	81.11
**at 25 × 25 pixels**	**at 25 × 25 pixels**	**73.80**
**at 80 × 80 pixels**	**at 25 × 25 pixels** (synthesizing masked face at 80 × 80 and resizing it to 25 × 25 pixels for face recognition)	**73.54**
